# Spectroscopic study on the interaction of pristine C_60_ and serum albumins in solution

**DOI:** 10.1186/1556-276X-7-433

**Published:** 2012-08-02

**Authors:** Shufang Liu, Yu Sui, Kai Guo, Zhijuan Yin, Xibao Gao

**Affiliations:** 1 School of Public Health, Shandong University, Jinan, 250012, People's Republic of China

**Keywords:** Bovine serum albumin, Fluorescence, Fullerene, Human serum albumin, Spectroscopy

## Abstract

The interaction of nanomaterials with biological macromolecules is an important foundation of the design and the biological safety assessments of nanomaterials. This work aims to investigate the interaction between pristine C_60_ and serum albumins (human serum albumin and bovine serum albumin) in solution. Stable aqueous dispersion of C_60_ was prepared by simple direct ultrasonic method and characterized by UV–vis spectrophotometry, transmission electronic microscopy and dynamic light scattering techniques, and spectroscopic methods (fluorescence spectroscopy, synchronous fluorescence spectroscopy and circular dichroism spectroscopy) were utilized for the investigation. It was found that the fluorescence of serum albumins could be quenched by C_60_ nanoparticles in a substantially similar way. Slight changes of the surrounding microenvironment of amino residues were observed, while little effects on the protein secondary structure occurred. The different effects of dispersion methods on the interaction of C_60_ nanoparticles with serum protein were also compared and discussed.

## Background

Studies on the biological effects of nanomaterial are often focused on their cytotoxicity, deposits in target organs and ecological toxicology, while relatively less attention was paid to their interactions with biological macromolecules. Almost all the life processes are inseparable from the participation of biological macromolecules (including proteins and nucleic acids). Following the process of translocation across biological barriers, nanomaterials can be transported into the blood, combine with biological macromolecules in the blood and other tissues, and through the blood transport be deposited in target organs where they exert potential toxic effects [1-3]. Therefore, in-depth understanding of their interactions with biological macromolecules is not only one of the important contents of their potential biomedical applications such as drug delivery, but also related to some basic problems such as the existence of specific roles of the interactions and the biological safety issues of nanomaterials, and thus attracts extensive attention of research communities. However, research on this area is still in its infancy.

Fullerenes are one of the most popular carbon nanomaterials used in industrial manufacturing, synthesis, engineering and medicine. Previous studies already showed that fullerenes (mainly C_60_ and its derivatives) could exhibit certain cytotoxicity [4,5], penetrate the blood–brain barrier and be deposited in target organs such as brain, liver, spleen, etc. [6,7], and cause the DNA damage by oxidative stress [8]. In the research reported about interactions between fullerenes and protein, the majority concentrated in the protein adsorption to the surface of fullerenes and the binding location, while relatively less on the changes of protein conformation and structure [9-11]. Conformation and/or structure changes of a protein induced by being adsorbed to the surface of nanomaterials may result in the changes of its bioactivity, but so far direct evidence of these changes of proteins is sparse. In addition, due to the insoluble nature in water of pristine fullerenes, water-soluble modified fullerene derivatives were often used for the related research [12]. It has been reported that fullerene aqueous dispersions could be obtained by solvent displacement methods [13] or by the addition of surfactant [14] or polymer [15] to water. In the above methods, the organic solvent, surfactant or polymer may affect the accuracy of the experimental results. Among various biological macromolecules, as the most abundant protein in plasma, serum albumin has important physiological functions including the binding and transport of endogenous and exogenous substances. Study on the interaction between fullerenes and serum albumins might help us obtain some information of the transport and removal of fullerenes.

Based on the above, in this work, C_60_, one of the typical fullerenes, chosen representing nanomaterials, serum albumins (including human serum albumin (HSA) and bovine serum albumin (BSA)) served as subjects, the interaction between C60 and the two serum albumins was investigated by spectroscopic methods. The C_60_ aqueous dispersion was simply obtained by injecting C_60_ powder into water, followed by ultrasonic method, and characterized by UV–vis, transmission electron microscopy (TEM) and dynamic light scattering (DLS) techniques. Fluorescence spectroscopy, synchronous fluorescence spectroscopy and circular dichroism (CD) spectroscopy were utilized to investigate the interaction. The binding difference between C_60_ and two proteins, the location of C_60_ nanoparticles and the changes of protein conformation and secondary structure were also proposed.

## Methods

### Materials

The C_60_ powder (99.9% pure) was purchased from Puyang Yongxin Fullerene Technology Co. Ltd. (Henan, China) and used as received. HSA and BSA (free fatty acid fraction V) were purchased from Sigma-Aldrich (St. Louis, MO). PBS buffer premixed reagent (pH = 7.4) was obtained from Zhongshan Goldenbridge Biotechnology Co., LTD. (Beijing, China). All solutions were prepared with 18.3 MΩ distilled water (Millipore, USA). Care was taken to minimize exposure of the samples to light during the experiments.

### Instruments

The fluorescence and synchronous fluorescence spectra of proteins were recorded on a FL-4500 Spectrofluorimeter equipped with a 1.0 cm quartz cell. The CD spectra of proteins were recorded on a J-810 Circular Dichroism Spectrometer (Jasco, Japan). UV-1101 spectrophotometer (Shimadzu, Japan), H-7000 transmission electron microscopy (Hitachi, Japan) and BI-200SM/BI-9000 laser light scattering system (Brookhaven, USA) were used to characterize the C_60_ dispersion. All the measurements were done at room temperature.

### Preparation of C60 dispersion

The C_60_ dispersion was obtained using ultrasonic method. The C_60_ powder was dissolved in distilled water and sonicated for 48 h. Remove the upper dispersion and abandon the bottom dispersion which contained the undissolved C_60_ powder. The obtained C_60_ dispersion was stored in a 4°C refrigerator and dispersed by ultrasound for 30 min before using.

### UV–vis absorption spectroscopic measurements

The UV–vis absorption spectra were recorded on a UV-1101 UV–vis recording spectrophotometer (Shimaza, Tokyo, Japan) with a 1.0 cm quartz cell.

### Dynamic light scattering measurement

The DLS measurement was performed on a BI-200SM/BI-9000 laser light scattering system (Brookhaven, USA) with a 1.0 cm quartz cell. The measurement was done at room temperature.

### Transmission electron microscopic observation

Specimen for TEM observation was prepared by depositing a drop of C_60_ dispersion on a 150-mesh copper gird and then air-dried in the dark. The TEM image of nC_60_ nanoparticles was taken from H-7000 transmission electron microscopy (Hitachi).

### Fluorescence spectra measurements of proteins

The protein fluorescence measurements were recorded with a FL-4500 spectrofluorimeter in a 1 cm quartz cuvette. The excitation and emission slit width (each 2.5 nm) and scan speed (1200 nm/min) were kept constant for all experiments.

### Synchronous fluorescence spectra measurements of proteins

The synchronous fluorescence measurements of proteins were recorded with a FL-4500 spectrofluorimeter in a 1.0 cm quartz cuvette and the excitation and emission slit width were both 2.5 nm with a scan speed of 1200 nm/min. A constant wavelength interval of Δλ = 20 nm or Δλ = 60 nm was set for the measurements.

### Circular dichroism spectroscopic measurements of proteins

The CD spectra of proteins were recorded with JASCO-J810 in a 1 mm quartz cuvette with a scan speed of 600 nm/min.

## Results and discussion

### The UV–vis spectra of C_60_ dispersions by different preparation methods

**Figure 1:**
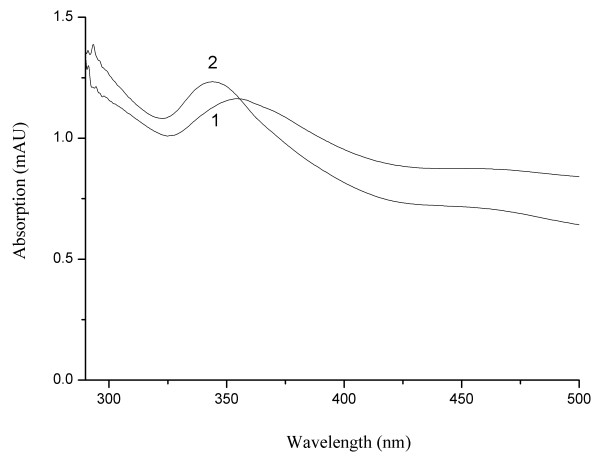
**The UV–vis absorption of C**_**60**_**dispersions.** (**1**). Directly dissolved in water, (**2**). Prepared by solvent replacement.

The C_60_ dispersion used in the following experiments was prepared by direct ultrasonic method and characterized by UV–vis spectrophotometric method. In order to comprehend the impact of preparation method on the C_60_ dispersion, we also prepared C_60_ dispersion by solvent displacement method (The solution of C_60_ in toluene was injected into water and dispersed by ultrasound. The toluene was allowed to evaporate under vacuum.) described according to the report [13], and their UV–vis absorption spectra were shown in Figure 1.

As shown in Figure 1, there was obvious difference between the absorption spectra of C_60_ dispersions prepared by the two different methods. For C_60_ dispersion prepared by direct ultrasonic method (Figure 1, line 1), it had absorption in the range of 325–440 nm with a maximum absorption wavelength of 355 nm, while C_60_ dispersion prepared by solvent replacement (Figure 1, line 2) had absorption in the range of 320–440 nm with a maximum absorption wavelength of 344 nm. The shift of the absorption peak of C_60_ nanoparticles might result from the different morphology and particle size of C_60_ nanoparticles. The concentration of the prepared C_60_ dispersion (prepared by direct ultrasonic method) was quantitated by UV–vis spectrophotometry at 344 nm and its concentration was 1.39 × 10^-5^ mol/L.

### Characterization of fullerene dispersion

**Figure 2:**
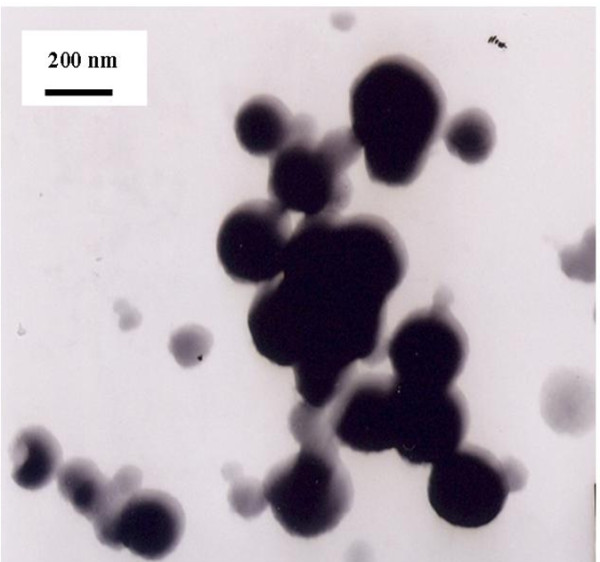
**TEM micrograph showing the C**_**60**_**clusters in an aggregated state.**

**Figure 3:**
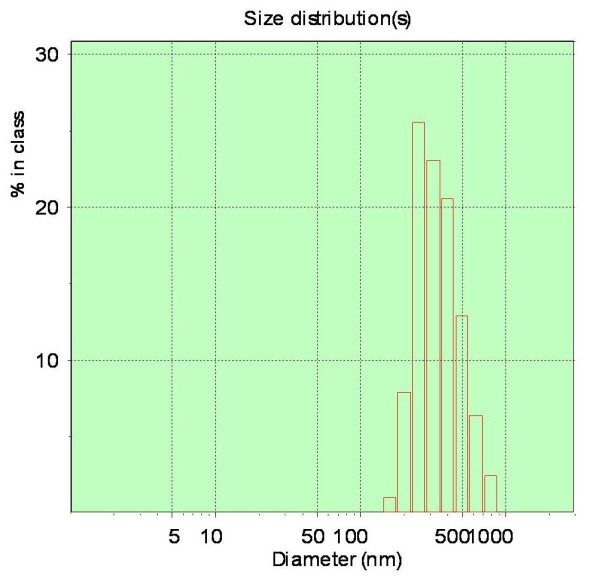
**The DLS spectrum of C**_**60**_**dispersion.**

Dynamic light scattering (DLS) is a technique for measuring the hydrodynamic size distribution of molecules and submicron particles, while TEM can directly offer the shape and size information of submicron particles in dry state. Due to the different principles, specimen characterized by these two methods sometimes may have different size distributions, and thus the combination of them is usually taken. In this work, the size distribution of nC_60_ nanoparticles in prepared C_60_ dispersion was measured by DLS and TEM techniques and the results were shown in Figure 2 and Figure 3.

The TEM image showed C_60_ nanoparticles were round-like and in the range of 120–260 nm, which agreed well with the average hydrodynamic diameter measured by DLS (The DLS spectrum showed C_60_ nanoparticles were mainly in the range of 160–500 nm with an average particle size of 283 nm.), and was significantly larger than the values reported previous [13,16]. The size distribution difference of C_60_ nanoparticles might be a possible explanation for the difference between the UV–vis absorption spectra in Figure 1.

### Quenching of protein fluorescence by C_60_ nanoparticles

**Figure 4:**
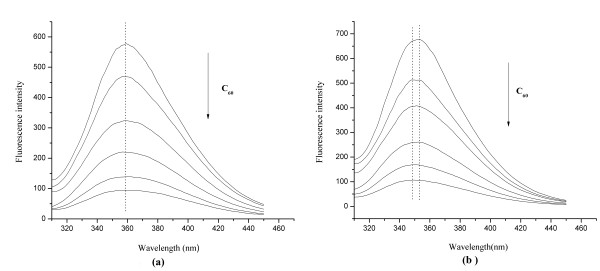
**The fluorescence spectra of HSA (a) and BSA (b) in the absence and presence of C**_**60**_**nanoparticles.** Conditions: HSA/BSA: 1.0 × 10^-5^ mol/L; C_60_ (from up to down): 0, 1.39, 2.78, 5.56, 8.34, 11.12 × 10^-6^ mol/L; pH = 7.4; ex = 292 nm.

As the most abundant protein in plasma, serum albumin (especially HSA and BSA) is frequently used in biophysical and biochemical studies. From the fluorescence change of HSA and BSA, people could get some information of interactions between protein and exogenous substances. Figure 4 showed the changes of HSA and BSA fluorescence in the absence and presence of C_60_ nanoparticles. Under 292 nm excitation, both BSA and HSA emitted fluorescence in the range of 300–450 nm with a maximum wavelength of 352 nm, which could be quenched by C_60_ nanoparticles with a dose-effect relationship. When the concentration of C_60_ nanoparticles increased to 11.12 × 10^-6^ mol/L, the maximum emission wavelength of BSA showed a blue-shift of about 4 nm, which indicated a slight change of the microenvironment polarity around the tryptophan residues and/or tyrosine residues occurred, and they were exposed to a hydrophobic environment [17]. No obvious shift of the maximum emission wavelength of HSA was observed, indicating C_60_ nanoparticles could not cause the change of HSA conformation.

**Figure 5:**
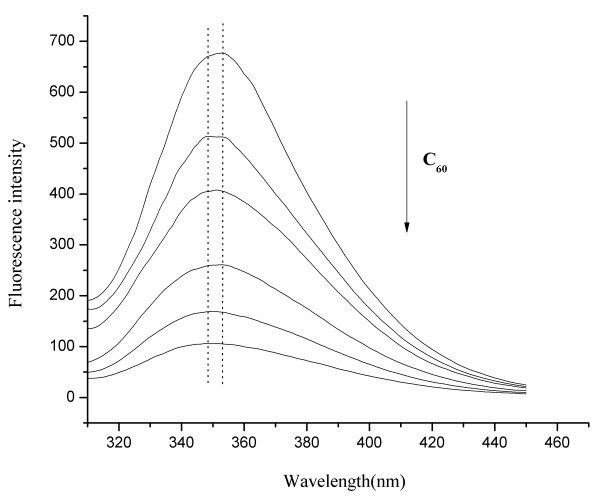
**Stern-Volmer plots of HSA and BSA fluorescence quenching by C**_**60**_**nanoparticles.** (F_0_: The fluorescence intensity of BSA/HSA in the absence of C_60_; F: The fluorescence intensity of BSA/HSA in the presence of C_60_).

In order to understand the difference between the interaction of C_60_ and two proteins, the Stern-Volmer curves [18] of HSA and BSA were plotted and the results were shown in Figure 5. The Stern-Volmer plots of HSA and BSA were similar, indicating C_60_ might have the similar interaction mode with HSA and BSA, which was consistent with the reported non-specific absorption of protein to C60 nanoparticles [10].

### The binding of HSA/BSA to C_60_ nanoparticles

**Figure 6:**
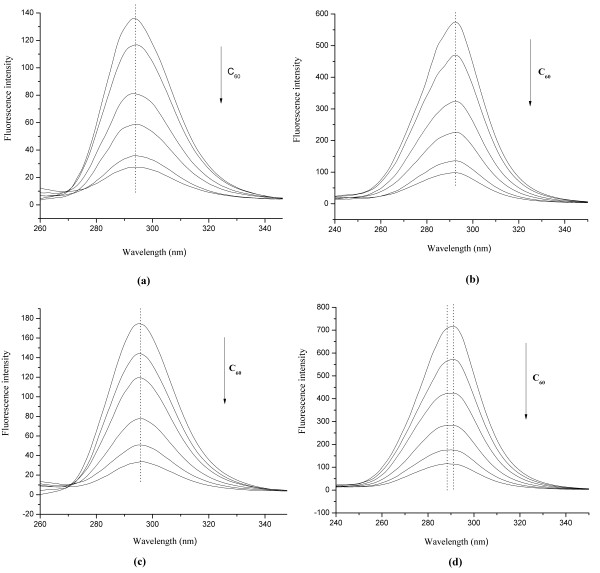
**The synchronous fluorescence spectra of HSA (a, b)and BSA (c, d) in the absence and presence of C**_**60**_**nanoparticles.** (**a.** HSA, Δλ = 20 nm; **b.** HSA, Δλ = 60 nm; **c.** BSA, Δλ = 20 nm; **d.** BSA, Δλ = 60 nm.) Conditions: HSA/BSA: 1.0 × 10^-5^ mol/L; C_60_ (from up to down): 0, 1.39, 2.78, 5.56, 8.34, 11.12 × 10^-6^ mol/L; pH = 7.4; ex = 292 nm.

The synchronous fluorescence spectroscopy has been widely applied to the study of conformational change in proteins. Fixed excitation and emission wavelength interval of Δλ = 20 nm mainly shows the spectral characteristics of tyrosine residues and Δλ = 60 nm shows spectral characteristics of the tryptophan residues [17]. For further comprehending the impact of C_60_ on HSA/BSA conformation and acquiring some information of C60 binding location, the synchronous fluorescence spectroscopy were used to characterize the binding of C_60_ to HSA/BSA, and the results were shown in Figure 6.

As can be seen from Figure 6, the fluorescence intensity of tryptophan residues was 3–4 times that of tyrosine residues, which means the HSA/BSA intrinsic fluorescence is mainly from tryptophan residues. The fluorescence intensity of tryptophan and tyrosine residues could be quenched by the addition of C60 nanoparticles with a dose-effect relationship. For BSA, the maximum emission wavelength of tryptophan residues had a slight blue-shift of about 3–4 nm, while the maximum emission wavelength of tyrosine residues remained basically unchanged, indicating the polarity decrease of the surrounding microenvironment of tryptophan residues. For HSA, consistent with the fluorescence quenching experiment (Figure 4), there was no peak wavelength shift of the synchronous fluorescence spectra. In the previous work, the C_60_ nanoparticles could cause red shift of maximum emission wavelength of tryptophan residues [13], which was different from this experiment, and we speculated that it might be due to the different size distribution of C_60_ nanoparticles resulted from the different preparation methods of C_60_ dispersions.

**Figure 7:**
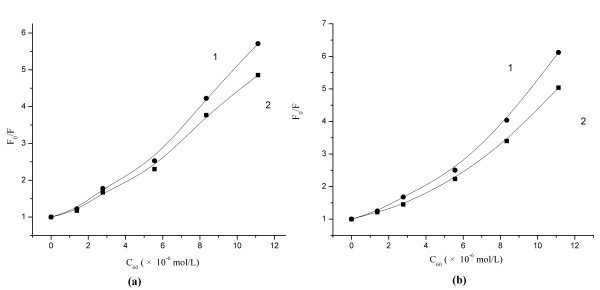
**Stern-Volmer plots of HSA (a) and BSA (b) synchronous fluorescence quenching by C**_**60**_**.** (1): Δλ=60 nm; (2): Δλ=20 nm.

For further understanding the binding location of C_60_ to HSA and BSA, the Stern-Volmer curves of tryptophan and tyrosine residues of HSA and BSA from the synchronous fluorescence spectra were plotted (Figure 7), and the results showed the fluorescence quenching effect of tryptophan residues by C_60_ was stronger than that of tyrosine residues, which indicated the binding site was located in the vicinity of tryptophan residues.

### The secondary structure changes of HSA and BSA

Circular dichroism spectroscopy is an important experimental technique for the study of protein conformation change, which is often used to investigate the secondary and the tertiary structure of proteins. The CD spectra of proteins are generally divided into two wavelength ranges, the far-UV circular dichroism (178–250 nm) and near-UV circular dichroism (250–320 nm). The far-UV region CD spectrum reflects the circular dichroism of peptide bond. The absorption peaks of CD spectra generated from proteins or peptides with different secondary structures were different from each other. By measuring the far-UV CD spectra of proteins or peptides, people could get some information of protein secondary structures, among which 208 nm and 222 nm are the characteristic peaks of α-helix structure [19,20].

**Figure 8:**
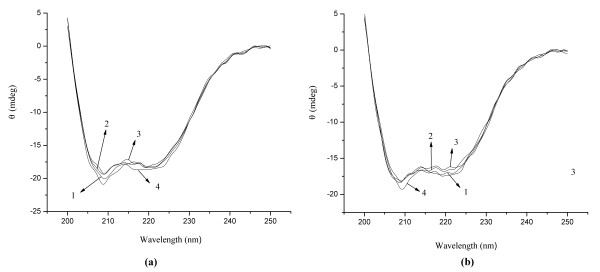
**The CD spectra of HSA (a) and BSA (b).** Conditions: HSA/BSA: 1.0 × 10^-6^ mol/L; C_60_: (1 to 4): 0, 2.78, 5.56, 11.12 × 10^-6^ mol/L; pH = 7.4.

In this experiment, the CD spectra of HSA and BSA in the absence and presence of different concentrations of C_60_ nanoparticles were measured and their α-helix and β-sheet structure contents were calculated utilizing the equipped calculation software of Yang’s equation, and the results were shown in Figure 8 and Table 1.

**Table 1:** The α-helix and β-sheet structure contents of HSA and BSA

**C**_**60**_	**BSA**		**HSA**	
**(×10**^-6^** mol/L)**	**α-helix**	**β-sheet**	**α-helix**	**β-sheet**
0	28.9%	26.7%	27.6%	26.3%
2.78	27.8%	26.6%	28.2%	27.8%
5.56	29.0%	26.6%	27.7%	29.4%
11.12	28.6%	25.9%	26.6%	26.4%

In the absence and presence of C_60_ nanoparticles. BSA, bovine serum albumin; HSA human serum albumin.

Figure 8 showed the CD spectra of HSA and BSA in the absence and presence of C_60_ nanoparticles. With the addition of C_60_ dispersion, the absorption of HSA and BSA at 208 nm and 222 nm had slight changes, but no dose–response relationship was observed, which was consistent with the no-significant-difference contents of α-helix and β-sheet structure of HSA and BSA in the absence and presence of C_60_ nanoparticles (Table 1). In the previous work, C_60_ nanoparticles (prepared by solvent replacement) could cause the α-helix content of HSA increased and the absorption of HSA and BSA at 208 nm and 222 nm weaker with a dose–response relationship [13]. The difference of the two different experimental results may be due to the different sizes of C_60_ nanoparticles, or residual toluene adsorbed on the surface of C_60_ nanoparticles in the latter experiment. The mechanism of the difference of protein structure and conformation changes caused by the two prepared C60 nanoparticles need further detailed study.

## Conclusions

C_60_ could be dispersed in water by ultrasonic method as fine clusters. The fluorescence of HSA and BSA could be quenched by C60 nanoparticles by non-specific adsorption. C_60_ nanoparticles mainly located in the vicinity of tryptophan residues, and caused slight changes of their surrounding microenvironment (only for BSA). During the adsorption process, no obvious changes of the secondary structure of HSA/BSA were observed. Compared with C_60_ dispersion by solvent exchange method, the C_60_ dispersion used in this work has little effect on the protein structure, which prompted us to conclude that dispersion method would be important for the bio-safety study of nanomaterials including fullerenes. Direct ultrasonic method can effectivlely avoid the interference of solvents or solubilizer, and thus more accurate for the assessement of the potential toxicity of fullerenes.

## Competing interests

The authors declare that they have no competing interests.

## Authors' contributions

XG conceived and designed the experiment. YS and KG performed the experiment and analyzed the data. ZY contributed to materials and analysis. SL wrote the paper. All authors read and approved the final manuscript.
